# Supplementary material for: Causal Relationship between Aging and Anorexia Nervosa: A White-Matter-Microstructure-Mediated Mendelian Randomization Analysis
Source: Biomedicines. 2024 Aug 16;12(8):1874. doi: 10.3390/biomedicines12081874 (PMC11351342; doi:10.3390/biomedicines12081874)
Supplement: Supplementary file 1 [file biomedicines-12-01874-s001.zip › Supplementary Figure/Figure S4/Singe SNP plot of MR analysis for TL to ubm-b-1947.pdf]

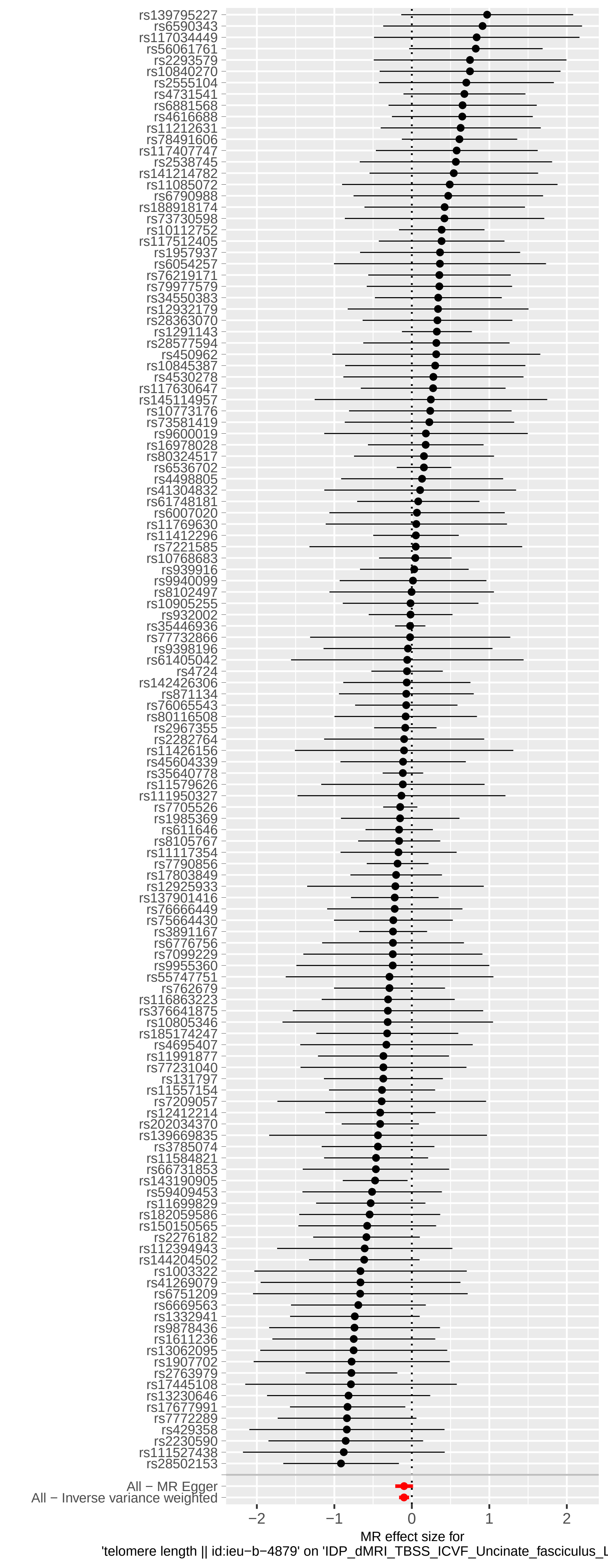
rs139795227
rs6590343
rs117034449
rs56061761
rs2293579
rs10840270
rs2555104
rs4731541
rs6881568
rs4616688
rs11212631
rs78491606
rs117407747
rs2538745
rs141214782
rs11085072
rs6790988
rs188918174
rs73730598
rs10112752
rs117512405
rs1957937
rs6054257
rs76219171
rs79977579
rs34550383
rs12932179
rs28363070
rs1291143
rs28577594
rs450962
rs10845387
rs4530278
rs117630647
rs145114957
rs10773176
rs73581419
rs9600019
rs16978028
rs80324517
rs6536702
rs4498805
rs41304832
rs61748181
rs6007020
rs11769630
rs11412296
rs7221585
rs10768683
rs939916
rs9940099
rs8102497
rs10905255
rs932002
rs35446936
rs77732866
rs9398196
rs61405042
rs4724
rs142426306
rs871134
rs76065543
rs80116508
rs2967355
rs2282764
rs11426156
rs45604339
rs35640778
rs11579626
rs111950327
rs7705526
rs1985369
rs611646
rs8105767
rs11117354
rs7790856
rs17803849
rs12925933
rs137901416
rs76666449
rs75664430
rs3891167
rs6776756
rs7099229
rs9955360
rs55747751
rs762679
rs116863223
rs376641875
rs10805346
rs185174247
rs4695407
rs11991877
rs77231040
rs131797
rs11557154
rs7209057
rs12412214
rs202034370
rs139669835
rs3785074
rs11584821
rs66731853
rs143190905
rs59409453
rs11699829
rs182059586
rs150150565
rs2276182
rs112394943
rs144204502
rs1003322
rs41269079
rs6751209
rs6669563
rs1332941
rs9878436
rs1611236
rs13062095
rs1907702
rs2763979
rs17445108
rs13230646
rs17677991
rs7772289
rs429358
rs2230590
rs111527438
rs28502153
All − MR Egger
All − Inverse variance weighted
−2
−1
0
1
2
MR effect size for
'telomere length || id:ieu−b−4879' on 'IDP_dMRI_TBSS_ICVF_Uncinate_fasciculus_L
